# Artificial intelligence in the GPs office: a retrospective study on diagnostic accuracy

**DOI:** 10.1080/02813432.2021.1973255

**Published:** 2021-09-29

**Authors:** Steindor Ellertsson, Hrafn Loftsson, Emil L. Sigurdsson

**Affiliations:** a Primary Health Care Service of the Capital Area, Reykjavik, Iceland; b Department of Computer Science, Reykjavik University, Reykjavik, Iceland; c Development Centre for Primary Health Care in Iceland, Reykjavik, Iceland; d Department of Family Medicine, University of Iceland, Reykjavik, Iceland

**Keywords:** General practice, artificial intelligence, primary headache disorders, electronic health records, statistical data interpretation, computer-assisted diagnosis, Iceland

## Abstract

**Objective:**

Machine learning (ML) is expected to play an increasing role within primary health care (PHC) in coming years. No peer-reviewed studies exist that evaluate the diagnostic accuracy of ML models compared to general practitioners (GPs). The aim of this study was to evaluate the diagnostic accuracy of an ML classifier on primary headache diagnoses in PHC, compare its performance to GPs, and examine the most impactful signs and symptoms when making a prediction.

**Design:**

A retrospective study on diagnostic accuracy, using electronic health records from the database of the Primary Health Care Service of the Capital Area (PHCCA) in Iceland.

**Setting:**

Fifteen primary health care centers of the PHCCA.

**Subjects:**

All patients that consulted a physician, from 1 January 2006 to 30 April 2020, and received one of the selected diagnoses.

**Main outcome measures:**

Sensitivity, Specificity, Positive Predictive Value, Matthews Correlation Coefficient, Receiver Operating Characteristic (ROC) curve, and Area under the ROC curve (AUROC) score for primary headache diagnoses, as well as Shapley Additive Explanations (SHAP) values of the ML classifier.

**Results:**

The classifier outperformed the GPs on all metrics except specificity. The SHAP values indicate that the classifier uses the same signs and symptoms (features) as a physician would, when distinguishing between headache diagnoses.

**Conclusion:**

In a retrospective comparison, the diagnostic accuracy of the ML classifier for primary headache diagnoses is superior to GPs. According to SHAP values, the ML classifier relies on the same signs and symptoms as a physician when making a diagnostic prediction.

**Keypoints**
- Little is known about the diagnostic accuracy of machine learning (ML) in the context of primary health care, despite its considerable potential to aid in clinical work. This novel research sheds light on the diagnostic accuracy of ML in a clinical context, as well as the interpretation of its predictions. If the vast potential of ML is to be utilized in primary health care, its performance, safety, and inner workings need to be understood by clinicians.

## Introduction

On a typical day, general practitioners (GPs) make multiple decisions when diagnosing and treating patients. They have limited access to immediate imaging diagnostics and tests and rely more on the patient's history and clinical examination than the second and tertiary stages of healthcare. To establish a diagnosis, a GP starts with the chief complaint, makes a hypothesis with a perceptual list of differential diagnoses, and asks the patient a series of targeted questions to include or exclude diagnoses. The GP then performs a clinical examination to confirm further or refute diagnoses while deciding if further diagnostic tests are needed. When the GP has reached a diagnostic conclusion, with a reasonable degree of certainty, he makes the diagnosis. In light of this description, a part of a GP's role requires him to act as a classifier of diseases. Physicians store their reasoning process in a free text format, often referred to as the clinical text note (CTN), which is a part of a patient's electronic health record (EHR). We conjecture that the CTNs can be used to develop predictive models for any step in the clinical decision-making process.

With the increased adoption of EHRs and thus, data availability, methods based on machine learning (ML) have become a focus of research in health care. ML has been shown to be a powerful tool in the medical diagnostic process with broad future possibilities in general practice [1]. ML has matched or exceeded human performance in multiple visual diagnostic tasks, such as diagnosing diabetic retinopathy from fundoscopy images [2,3], diagnosing radiology images [4,5], diagnosing skin lesions [6,7], and interpreting electrocardiograms [8,9]. However, the use and research of ML in a medical context is still in its early stages [10], with surveys showing less than 10% of healthcare providers using ML to assist in daily clinical work in 2019 in Britain [11].

The literature on peer-reviewed studies comparing the diagnostic accuracy of ML classifiers to GPs is lacking. The aim of this study was to explore the performance of an ML classifier on a common clinical problem in general practice. The null hypothesis put forward was that an ML classifier could not match or outperform the diagnostic accuracy of GPs on primary headache diagnoses. Headaches account for up to five percent [12] of all clinical problems in general practice (the majority of which are primary headaches) and are often a tough clinical problem to diagnose correctly. Primary headache diagnoses are made predominantly in primary care [12], and since the input data is extracted from CTNs, written by GPs, we chose GPs as benchmark. The four most common primary headache diagnoses were selected: tension-type headache (TTH), migraine with aura (MA+), migraine without aura (MA-), and cluster headache (CH). Interpretability is essential in medical settings [13], and the results of the examination of the intricacies of diagnostic ML models are scarcely published in the medical literature. Therefore, the impact of each clinical feature to the model's predictions was scrutinized with Shapley Additive Explanations (SHAP), which are widely used within the field of AI research to interpret ML models [14].

## Materials and methods

We conducted a retrospective study and obtained 15,024 CTNs from 13,682 patient visits to one of 15 clinics of the Primary Health Care of the Capital Area (PHCCA), the largest provider of primary health care (PHC) in Iceland. As an inclusion criterion, we selected every PHC consultation where one of the four headache diagnoses was made, from 1 January 2006 to 30 April 2020. A large portion of the dataset included CTNs that contained little clinical information. Such information-scarce notes were filtered out by creating a medical keyword dictionary, containing the relevant symptoms a physician asks about when making each diagnosis. The dictionary contained words that physicians use to describe different headache symptoms in text format, for example, nausea, phonophobia, vomiting, etc. and was created by manually reading a subsample of the dataset (around 1% for each class) and selecting the right keywords. The dictionary was then applied to the whole dataset, filtering out CTNs without the chosen keywords, resembling methodology used in a similar research study [15]. Simultaneously, 93 duplicate CTNs were removed, leaving us with 2,563 information-rich CTNs, or roughly 17% of the original dataset. These CTNs had an accompanying headache diagnosis that falls into one of the four headache diagnosis categories mentioned above.

Eight hundred randomly selected CTNs were then manually annotated by a physician. The annotation method was inspired by a group of researchers who applied similar annotations on medical text [15] and its purpose is to assign binary and numerical values to clinical features, representing the existence or lack of specific signs and symptoms in the CTNs, as they are textually referenced in the CTNs. As an example of annotation, consider a typical, frequently seen sentence in a CTNs: ‘The patient experienced photophobia but no phonophobia’. The questions, ‘Does the patient have photophobia?’ and ‘Does the patient have phonophobia?’ are annotated as two features, with binary values of 1 and 0. Numerical value features were assigned a value in a specific range, for example, a feature for blood pressure (e.g. with the value 134/78) or a feature for temperature (e.g. with the value 37.1 °C). This annotation process was repeated for every clinical feature referenced in the 800 CTNs. Every sign and symptom were annotated, including those unrelated to headache diagnoses to reduce the annotator's possible bias who, importantly, was also blind to the diagnoses. Where possible, a feature was only annotated as positive if the feature was considered abnormal. To give an example, a question about tactile sensation in a patient's lower extremities, was phrased as ‘Does the patient have signs of reduced and/or asymmetric tactile sensation in the lower extremities?’, instead of ‘Does the patient have normal and/or symmetric tactile sensation in the lower extremities?’ Therefore, if a patient had abnormal tactile sensation, the binary value of 1 was assigned, and 0 if it was normal.

The annotation resulted in a dataset of 8,595 question-answer pairs (where a clinical feature is the question and the corresponding value is the answer), resulting in 254 different clinical features. This dataset was randomly split into training, validation, and test sets. The split was 75% (training), 12.5% (validation), and 12.5% (test) or 600, 100, and 100 CTNs in each, respectively. Thereafter, the ML classifier was trained on the training set, and the impact of each input feature examined using SHAP values. SHAP values are calculated by knocking out every feature of the input data, one at a time, and measuring how the predictions of the classifier change. SHAP values approximate the contribution of each feature to the model predictions, effectively revealing which features the ML classifier considers most important when making a prediction. A randomized grid search was performed to optimize the hyperparameters of the ML classifier before training, creating a four-class multi-classifier (one class for each of the four headache diagnoses) of type Random Forest. The validation set was used to further finetune the hyperparameters of the ML classifier. Once training was finished, the classifier's performance was compared to two groups of physicians: three GP specialists and three physician trainees in GP. The comparison was carried out using the test set, which had been set aside for this purpose, and to provide an unbiased evaluation of the final model fit on the training set. The physician validation process was performed via a custom web interface where the physicians reviewed the annotated features and their value for each CTN and subsequently selected one of the four headache diagnoses they found to be appropriate. Thus, the performance comparison was performed on the original diagnoses, to see how the ML classifier and the physicians compare when given the same set of information. A 10-fold cross-validation scheme was used to validate the results and to calculate 95% confidence intervals.

### Statistical analysis

The metrics used for the performance assessment were the following: sensitivity, specificity, Positive Predictive Value (PPV), Matthews Correlation Coefficient (MCC), Receiver Operating Characteristics (ROC) curve, and Area under the ROC curve (AUROC score). A confusion matrix is a statistical presentation of the performance of a classifier and consists of four quadrants. Each quadrant contains a value count for one of four performance variables: true-positive count (TP), false-positive count (FP), true-negative count (TN) and false-negative count (FN). The MCC is a useful metric for imbalanced datasets since it only produces a high score if the prediction obtained good results in all of the four quadrants of the confusion matrix. It ranges from −1 to 1, where 1 represents a perfect prediction. The MCC is defined as follows:

$$\mathrm{MCC}=\frac{\left(TP\;\times \;TN)-(FP\;\times \;FN\right)}{\sqrt{\left(TP+FP)(TP+FN)(FN+FP)(TN+FN\right)}}$$

We calculated the MCC for each diagnosis as a dichotomous outcome. We obtained an AUROC score by plotting the true-positive rate (TPR) against the false-positive rate (FPR) for each diagnosis at varying thresholds and calculating the area under the curve. The ROC curve was plotted for each diagnosis as a dichotomous outcome. All means are weighted if not stated otherwise. All data analyses were done in Python (version 3.6), in which the ML classifier was trained and validated with the scikit-learn library (version 0.22.1) [16].

## Results

**Figure 1.**
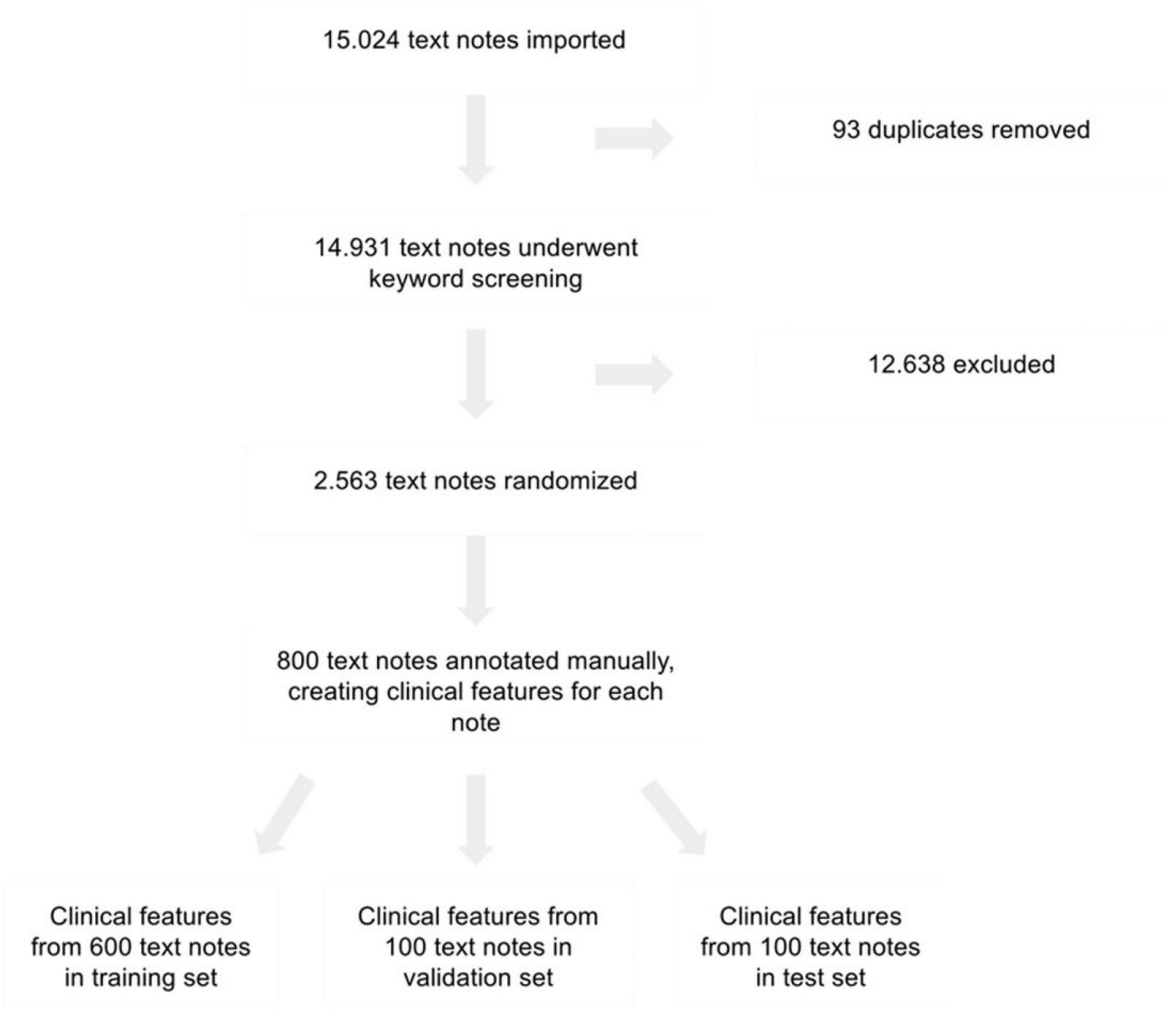
The inclusion and filtering process of the CTNs. The clinical features were created by annotating the CTNs, which were then split into training, validation and test sets.

The flow of participants is shown in Figure 1. A total of 12,368 notes were deemed as information-scarce and filtered out. 4,913 positive features and 3,682 negative features were annotated for a total of 8,595 features, resulting in 1.33 positive features for every negative one and 10.74 features in each CTN on average. Table 1 shows the demographic distribution of the training, validation and test datasets, which all have similar demographics and have women as the majority of patients. Table 2 shows the results and comparison of the classifier and physicians on the test set. The classifier outperforms all physicians for all metrics, except for specificity. The classifier's lower specificity for TTH draws its weighted average down as it achieves equal or higher specificity compared to the physicians on the other three diagnoses. The classifier achieves the best performance in the cases of TTH, MA+, and CH. It has the lowest performance for MA- as it, in some cases, struggles to discern between MA- and TTH.

**Table 1.** The demographic comparison of the training, validation and test set.

	Training set	Validation set	Test set
Total size	600	100	100
Female	434 (72.3%)	65 (65%)	72 (72%)
Mean age (min–max)	34.56 (6–90)	33.53 (8–78)	31.29 (8–77)

**Table 2.** The performance metrics for the classifier and physicians on the test set.

	ML classifier			
	Sensitivity	Specificity	PPV	MCC
Cluster headache	0.83	1.00	1.00	0.91
Migraine with aura	0.92	0.99	0.92	0.90
Migraine without aura	0.67	0.99	0.86	0.73
Tension headache	0.99	0.85	0.95	0.87
Weighted average	0.95	0.88	0.94	0.86
	GP Specialist 1			
Cluster headache	0.83	1.00	1.00	0.91
Migraine with aura	0.92	0.95	0.73	0.79
Migraine without aura	0.80	0.92	0.53	0.61
Tension headache	0.89	0.96	0.98	0.79
Weighted average	0.88	0.95	0.88	0.77
	GP Specialist 2			
Cluster headache	0.67	1.00	1.00	0.81
Migraine with aura	0.58	0.96	0.70	0.59
Migraine without aura	0.90	0.87	0.45	0.58
Tension headache	0.90	0.95	0.98	0.79
Weighted average	0.86	0.94	0.85	0.73
	GP Specialist 3			
Cluster headache	0.83	1.00	1.00	0.91
Migraine with aura	0.67	0.99	0.89	0.74
Migraine without aura	0.50	0.95	0.56	0.48
Tension headache	0.97	0.72	0.91	0.75
Weighted average	0.90	0.78	0.88	0.73
	GP Trainee 1			
Cluster headache	0.83	1.00	1.00	0.91
Migraine with aura	0.92	0.99	0.92	0.90
Migraine without aura	0.70	0.93	0.54	0.56
Tension headache	0.93	0.88	0.96	0.80
Weighted average	0.89	0.91	0.90	0.78
	GP Trainee 2			
Cluster headache	0.83	0.95	0.56	0.65
Migraine with aura	0.42	0.99	0.83	0.55
Migraine without aura	0.80	0.79	0.31	0.41
Tension headache	0.81	0.95	0.98	0.64
Weighted average	0.78	0.91	0.76	0.58
	GP Trainee 3			
Cluster headache	0.83	0.98	0.71	0.75
Migraine with aura	0.50	0.99	0.86	0.62
Migraine without aura	0.70	0.90	0.44	0.49
Tension headache	0.94	0.90	0.97	0.82
Weighted average	0.87	0.91	0.86	0.75

PPV stands for Positive Predictive Value and MCC for Matthews Correlation Coefficient.

Figure 2 shows the top-20 most impactful features by SHAP values for each diagnosis. Figure 2(a) displays the features with a significant effect on a positive CH prediction. These features include CH's autonomic symptoms: ptosis, conjunctivitis, lacrimation, runny/stuffy nose accompanying a unilateral headache located around a single eye. Myalgia symptoms reduce CH's diagnostic likelihood, as does visual disturbance, often accompanying MA+. In Figure 2(b), we see that the two most impactful features of MA + are prodromal symptoms and visual disturbance, both the hallmarks of an aura. Unilaterality and photophobia positively impact a MA + prediction, but less since MA- also typically has these features. Aura can present itself as limb numbness, which also positively affects the prediction. In some CTNs, only the word aura was found, without a more detailed description of the presentation. In the case of MA-, displayed in Figure 2(c), we see a different SHAP profile than for MA+. The classifier uses prodromal symptoms and visual disturbance to distinguish between MA + and MA-. The SHAP profile describes a unilateral headache, with accompanying nausea, vomiting, photophobia, phonophobia, and without symptoms of myalgia. This description fits well with MA-. Figure 2(d) shows the SHAP values for TTH, where we see a pattern of absence of symptoms. Only myalgia symptoms affect the prediction positively.

**Figure 2.**
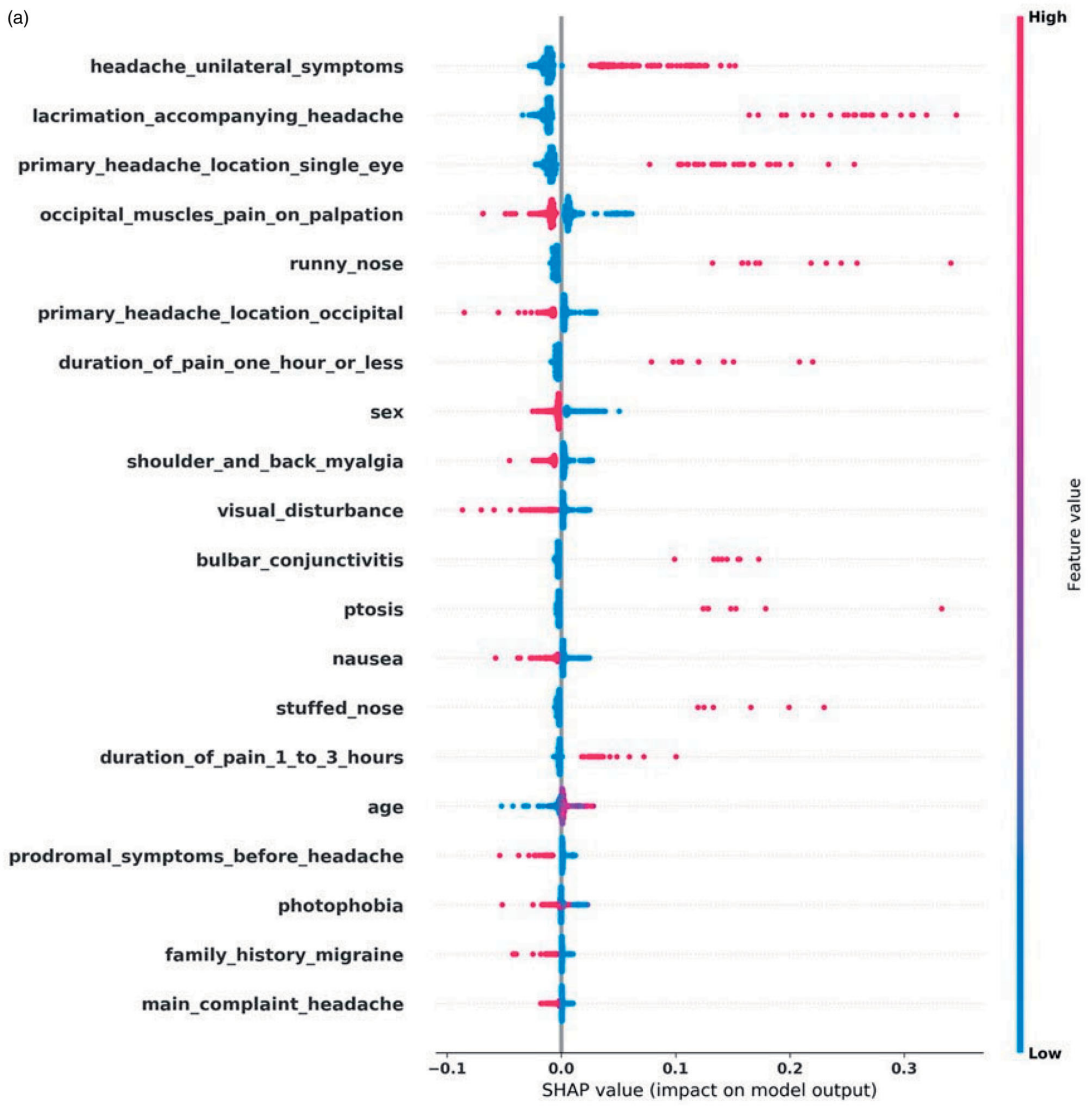
**(**a) Shapley Additive Explanations (SHAP) for the most impactful input features for a CH prediction. Each feature's name is on the left. The dots' color represents the feature's value, where blue color is lower than red. A blue dot means that a feature was negative in a single CTN, that is, it was not present. Red means the opposite. On the X-axis, the impact on the prediction is plotted, where higher score leads to increased probability of outputting a positive prediction.

**Figure 2.**
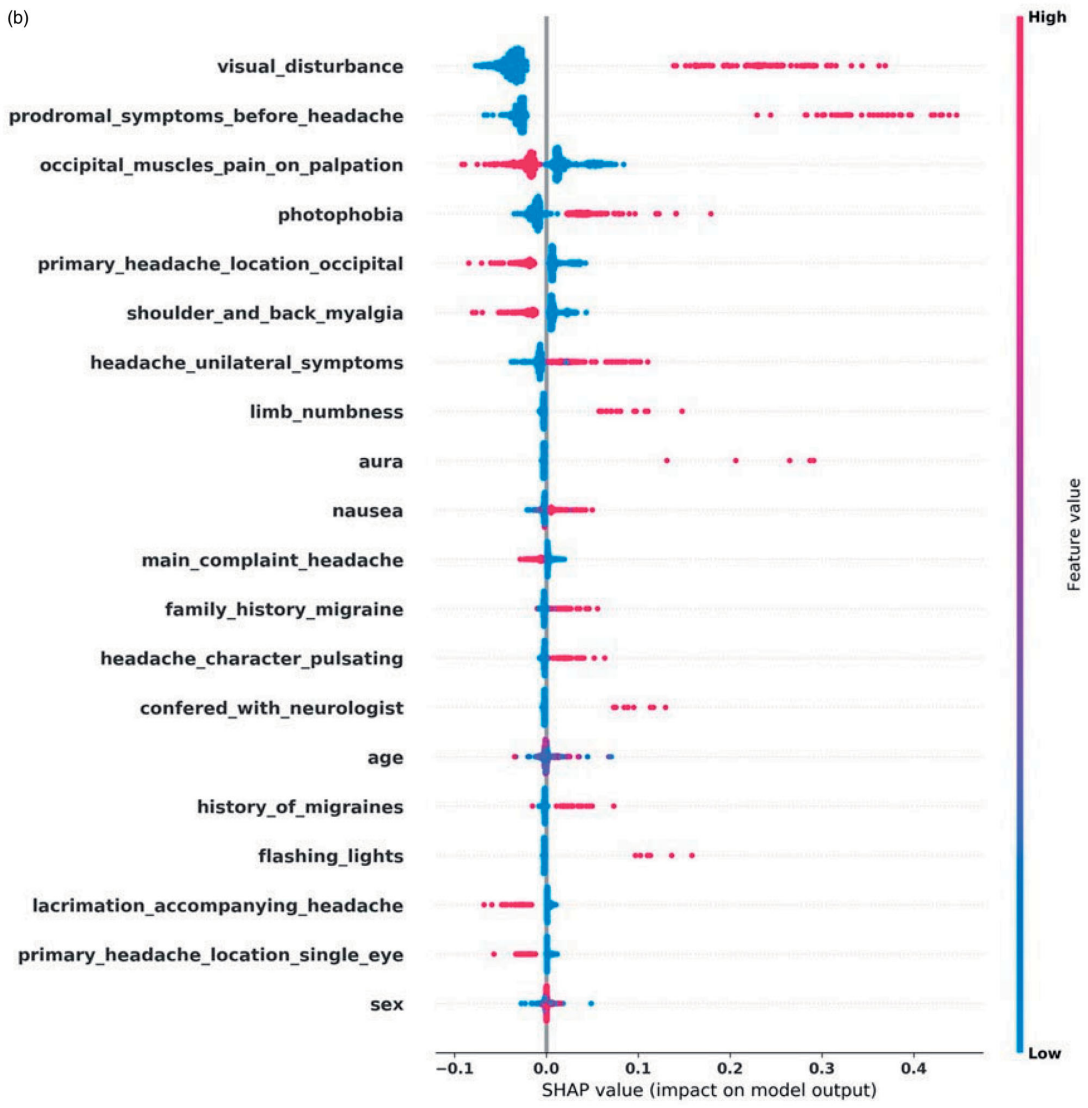
(b) Shapley Additive Explanations (SHAP) for the most impactful input features for an MA+prediction. Each feature’s name is on the left. The dots' color represents the feature’s value, where blue color is lower than red. A blue dot means that a feature was negative in a single CTN, that is, it was not present. Red means the opposite. On the X-axis, the impact on the prediction is plotted, where higher score leads to increased probability of outputting a positive prediction.

**Figure 2.**
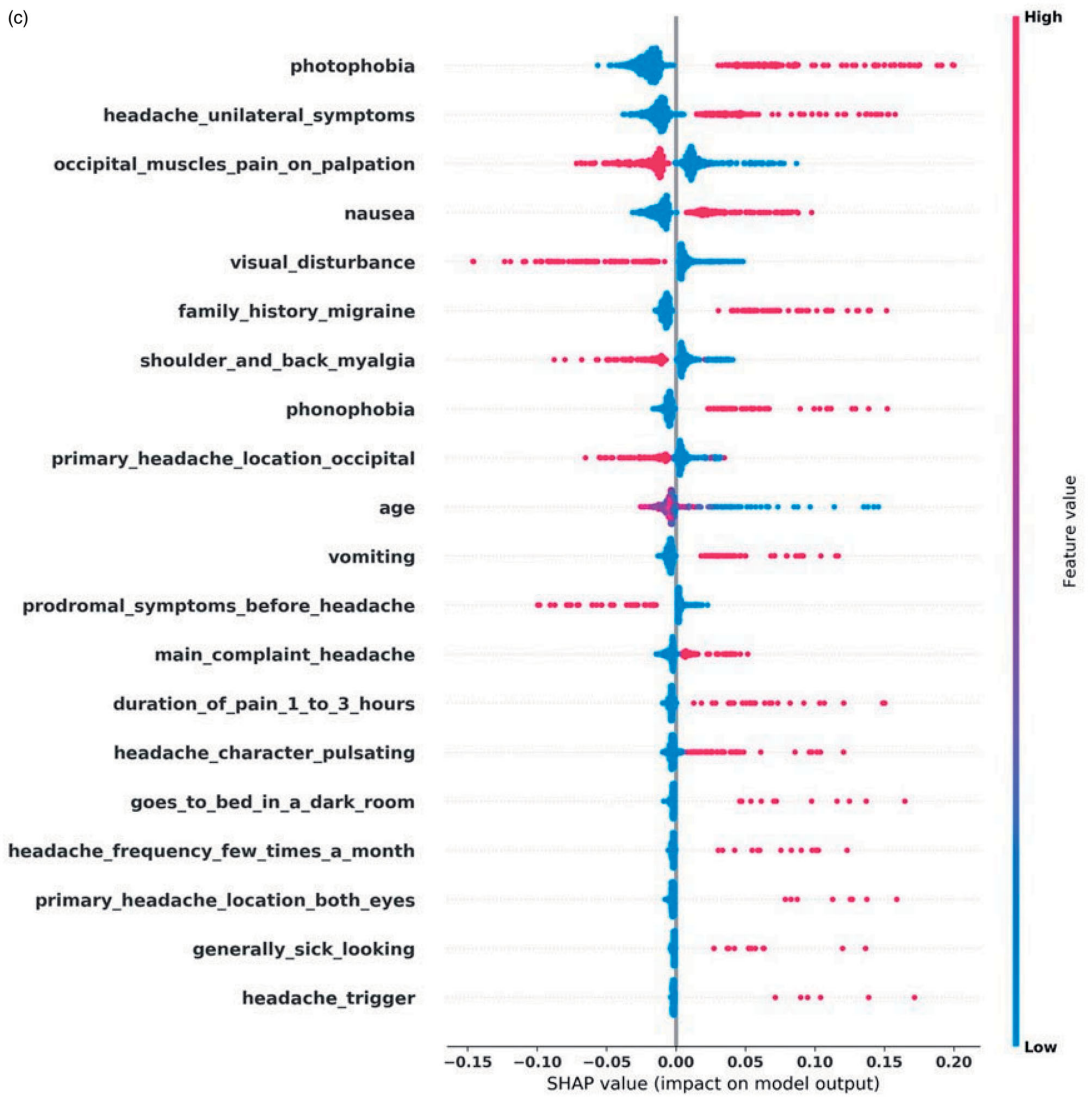
(c) Shapley Additive Explanations (SHAP) for the most impactful input features for an MA- prediction. Each feature’s name is on the left. The dots' color represents the feature’s value, where blue color is lower than red. A blue dot means that a feature was negative in a single CTN, that is, it was not present. Red means the opposite. On the X-axis, the impact on the prediction is plotted, where higher score leads to increased probability of outputting a positive prediction.

**Figure 2.**
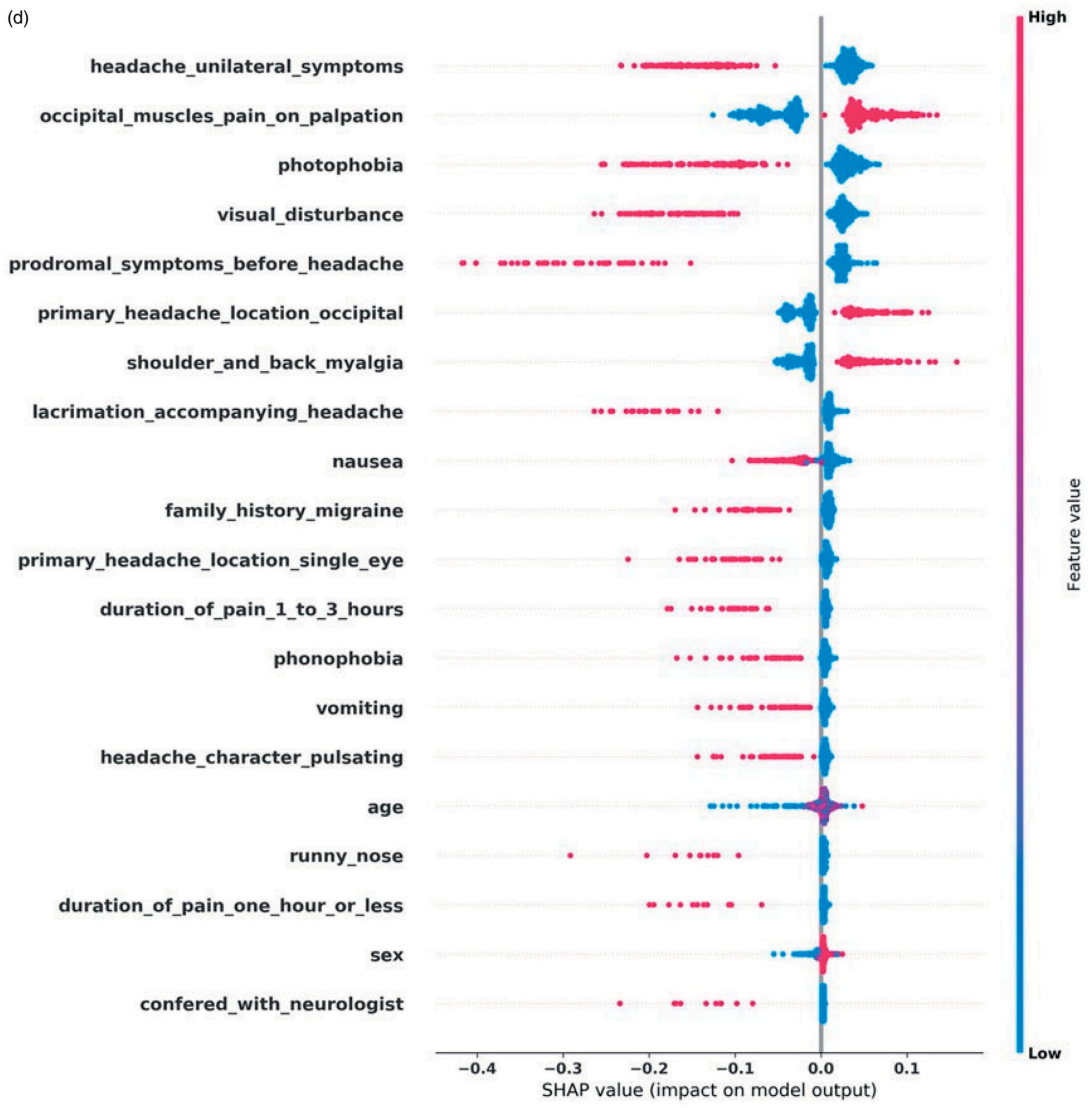
(d) Shapley Additive Explanations (SHAP) for the most impactful input features for a TTH prediction. Each feature’s name is on the left. The dots' color represents the feature's value, where blue color is lower than red. A blue dot means that a feature was negative in a single CTN, that is, it was not present. Red means the opposite. On the X-axis, the impact on the prediction is plotted, where higher score leads to increased probability of outputting a positive prediction.

**Figure 3.**
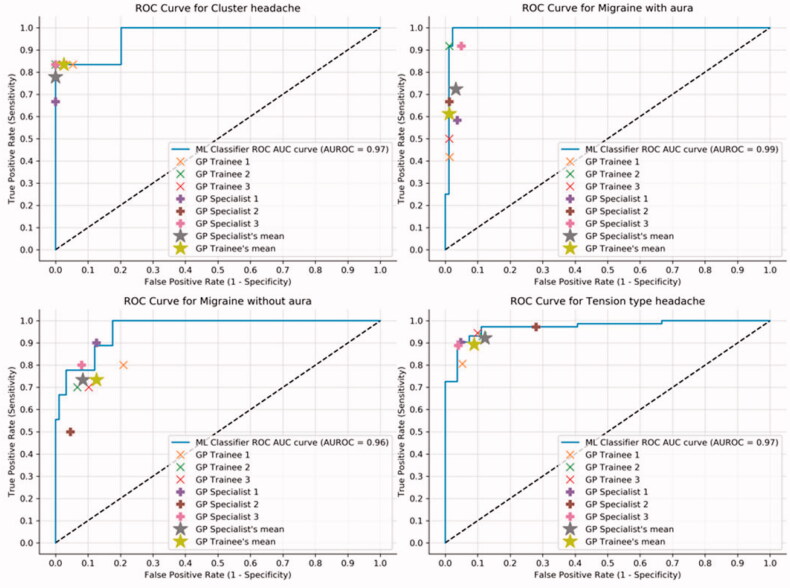
The ROC curve for the ML classifier, plotted for each diagnosis on the test set. The AUROC score is a measure of classifier performance with a maximum value of 1. It is calculated as the area under the ROC curve. The performance of each physician is plotted, as is the mean performance of the trainee physicians and specialists.

Figure 3 shows the ROC curve for the test set compared to the physicians. The ROC curve plots the true-positive rate (TPR) against the false-positive rate (FPR) and the performance of a classifier increases as the curve moves up and to the left. A perfect classifier covers the whole plot, achieving an AUROC score of 1. The mean results of the specialists and trainee physicians are located lower and to the right relative to the plotted line of the ML classifier on each plot, meaning the physicians achieve a lower ratio of TPR and FPR than the classifier on all diagnoses.

## Discussion

### Statement of principal findings

The ML classifier outperforms the physicians on all metrics except specificity. The MCC is arguably the most important metric, being the only one taking all four quadrants of the confusion matrix into account. The ROC curves in Figure 3 also shows a superior performance of the ML classifier compared to the physicians mean scores. In some cases, it struggles with discerning between TTH and MA- and the most probable explanation is that some patients in our dataset have an episodic TTH (ETTH), which often presents itself with mixed features of MA- and TTH. Physicians often misdiagnose these patients as having either a simple TTH or MA- [17,18], depending on the presentation of their symptoms. Additionally, the case count of MA- is relatively low. Interestingly, the classifier performs better in the case of CH, despite CH having the lowest case count. The difference is that CH has more distinguishing clinical features from the other three diagnoses, illustrating an important point: ML models are sensitive to the quality of the input data. If the training data contains errors and biases, the models learned will be erroneous and biased in the same manner. However, ML methods allow us to review the training data and correct for biases and diagnostic errors [19], which is impossible to do for physicians. As the quality of the training data increases, so will the performance of the ML models.

The SHAP profile for each diagnosis fits well with the symptoms and signs a physician would need to evaluate when suspecting these diagnoses. The SHAP profile of CH and the migraine diagnoses show features that affect the model positively, while in the case of TTH, the absence of symptoms is the main theme which fits well with the ill-defined clinical motif of TTH [17].

### Strengths and weaknesses of the study

Our study benefits from a large dataset and benchmarking against GPs, which is essential when validating clinical prediction models. The selection of a few diagnoses strengthens and limits the study, as the results are easier to interpret but lack a broader diagnostic application. The small sample size of non-TTH diagnoses, and the fact that the diagnoses are obtained from a single physician when training the ML classifier, poses a limitation. In future studies, it would be optimal to have multiple physicians agreeing on the diagnoses. Another limitation is that many physicians store only a part of their reasoning process in the CTNs. To counter this, roughly 84% of the CTNs were filtered out, leaving only information-rich CTNs before splitting the dataset into training, validation and test sets. A large dataset also reduces the effect of this limitation, since it is unlikely that all physicians leave out the exact same symptoms. However, the filtering does introduce a bias toward more complex clinical cases being included. A prospective study is needed to elucidate these limitations further. Since unmentioned clinical features were assigned a binary value of 0, and as physicians are more likely to register positive symptoms than negative, a clinical feature was only annotated as positive if it was considered abnormal, as mentioned before. The ML classifier was also trained with missing features marked specifically but the results were the same. Again, a prospective study could explore this possible limitation further. The annotation process also limits the study with a single GP-trainee annotating the dataset as resources did not allow for additional annotators. Having GPs auditing random subsamples of annotations would have increased the quality of the annotations.

### Findings in relation to other studies

We did not find any peer-reviewed papers on the evaluation of diagnostic ML classifiers in PHC settings in the literature, highlighting the importance of further research. Studies examining online symptom checkers (OSC) are the nearest comparison, as an ML classifier, as described above, would be implemented similarly. An audit study evaluated 23 symptom checkers across 770 standardized patient evaluations [20], reporting a significant variance in the OSCs' diagnostic performance, which was not close to matching the diagnostic performance of human physicians. Babylon Health, a PHC provider in Great Britain, has developed an OSC and reported comparable diagnostic accuracy to human physicians in a non-peer-reviewed paper [21]. The difference in the architecture of different OSCs complicates comparison, with many not disclosing their inner workings and research has shown that the design of computerized diagnostic system can have significant impact on their effectiveness [22].

### Meaning of the study

This research is a step toward developing accurate, safe, and interpretable ML classifiers that can positively impact PHC in multiple ways. The interpretability of clinical AI models is of essence, if clinicians are to integrate AI solutions into their workflow. If the results are generalizable, any arbitrary clinical prediction model could be developed, implemented as an online service. Such a service could potentially allow for pre-screening of patients, enable self-care and lead to a more correct use of the health care system, that is, patients using expensive emergency services less, and managing self-isolating symptoms at home [23,24].
